# The Effects of Brief Mindfulness Training on Attentional Processes: Mindfulness Increases Prepulse Facilitation but Not Prepulse Inhibition

**DOI:** 10.3389/fpsyg.2021.582057

**Published:** 2021-02-17

**Authors:** Ole Åsli, Marta F. Johansen, Ida Solhaug

**Affiliations:** Department of Psychology, University of Tromsø - The Arctic University of Norway, Tromsø, Norway

**Keywords:** startle, prepulse facilitation, mindfulness, attention, prepulse inhibition

## Abstract

Mindfulness is intentional focus of one’s attention on emotions, thoughts, or sensations occurring in the present moment with a nonjudgmental attitude. Recently there has been increased interest in the effects of mindfulness practice on psychological processes such as concentration, focus, and attention. In the present study, a prepulse inhibition/facilitation (PPI/PPF) paradigm was employed to investigate the effect of brief mindfulness practice on automatic attention regulation processes. PPI occurs when a relatively weak prepulse (e.g., a tone) is presented 30–500 ms before a startle-inducing stimulus, and reduces the magnitude of the startle response. Prepulse facilitation (PPF) is the increase in startle magnitude when the prepulse is presented 500 ms or more before the startle-eliciting stimulus. In the present study, the effect of engaging in a 23-min mindfulness exercise on PPI and PPF was investigated. Participants listened to either a mindfulness instruction (mindfulness group) or relaxing music (control group). In a PPI/PPF pretest and posttest, a startle-eliciting noise was presented at lead intervals of 60, 120, and 2,000 ms. Results showed that engaging in brief mindfulness practice increased prepulse facilitation at the 2,000 ms lead interval in the posttest compared to the pretest. The amount of PPI did not differ between tests.

## Introduction

Mindfulness is intentional focus of one’s attention on present-moment experience with a non-judgmental, accepting attitude. The disposition to be mindful is thought to increase with mindfulness training (MT), a form of meditation that has become more popular in recent years. Research has revealed that mindfulness training affects different parts of the human brain (Fox et al., 2014), and it seems to have a positive effect on both physical and psychological health (Goyal et al., 2014; Gotink et al., 2015). Clinical interventions, such as mindfulness based stress reduction (MBSR; Kabat-Zinn, 2003) and mindfulness based cognitive therapy (Teasdale et al., 1995), may be effective treatment methods to reduce symptoms associated with different physical and psychological disorders (Bohlmeijer et al., 2010; Khoury et al., 2013, 2015; Gu et al., 2015).

Recently there has been increased interest in the effects of mindfulness practice on psychological processes such as concentration, focus, and attention (Chiesa et al., 2011). Typically, this research has focused on three functionally and anatomically distinct subsystems of attention, termed *alerting*, *orienting*, and *conflict monitoring* (*executive functioning*). The Attention Network Test (ANT; Fan et al., 2002) measures the performance of the mentioned attentional components and is frequently used to investigate the effect of meditation on attentional abilities.

While research on the effect of meditation on conflict monitoring has reported enhanced performance in several studies (Wenk-Sormaz, 2005; Chan and Woollacott, 2007; Slagter et al., 2007; Moore and Malinowski, 2009; see Tang et al., 2015 for a review), the effects on alerting have been less clear. Studies investigating long-term meditators have found increased alerting, while most studies examining short-term effects of mindfulness meditation found no such effects (Tang et al., 2015).

Enhanced orienting has been reported in studies using both longer (3 months) and shorter periods of training. Jha et al. (2007) and MacCoon et al. (2014) found that participants in an 8-week mindfulness-based stress reduction (MBSR) course showed improved orienting post training in relation to the control group. Jha et al. (2007) found improvements in alerting only in experienced practitioners. Participants in a 1-month intensive mindfulness retreat improved alerting to exogenous stimulus detection in the posttest. Jha et al. (2007) related orienting to the term “concentrative attention,” and alerting was related to “receptive attention.” Further, they discussed that MT improves concentrative attention (orienting) first, and that improvements in receptive attention (alerting) develops slower and are therefore only present in expert practitioners.

Similarly, a systematic review by Alberto Chiesa et al. (2011) concluded that early phases of mindfulness training are associated with improvements in orienting (and conflict monitoring), whereas later phases seems to be associated with improved alerting. In sum, mindfulness appears to improve the attentional systems of orienting in the short term, while the system of alerting has been found to improve in long-term practitioners.

The startle reflex is an automatic reflex in response to intense, sudden stimuli. It consists of muscular activities including eyeblinks and contraction of muscles in the neck, shoulders, upper back, arms, and legs. In humans, it is often measured as electromyographic (EMG) activity of the orbicularis oculi, the muscle that closes the eyelids. Psychological processes can modify the amplitude, latency, and probability of the startle reflex. This is termed startle reflex modification. Prepulse inhibition (PPI; Graham, 1975) is one of the most studied forms of startle reflex modification. PPI occurs when a stimulus is presented shortly prior to the startle reflex-eliciting stimulus, and this inhibits the startle response.

As such, when a nonstartling stimulus (for instance, a tone) is presented just before a loud sudden (eyeblink-eliciting) noise, the startle reaction to the noise is diminished compared to the reaction to the noise alone. PPI typically occurs when the interval between prepulse (tone) and pulse (noise) is between 15 and 400 ms (Graham, 1975). PPI is thought to indicate the protection of processing of the weaker prepulse and is a measure of automatic attention. Research have found reduced PPI in numerous disorders including schizophrenia, obsessive–compulsive disorder, and panic disorder (Braff et al., 2001). Prepulse facilitation (PPF) is a related phenomenon, occurring either at very early intervals between pulse and prepulse or at later intervals, where the reaction to the second stimulus is larger. In this study, we were interested in PPF that occurs late, when the prestimulus is presented about 500–2,000 ms before the startle stimulus. Such late occurring PPF is thought to indicate a later stage of generalized orienting or attention (Wynn et al., 2004), i.e., an indication of orienting toward the prepulse (Graham, 1975). Reduced PPF have been reported in schizophrenia patients (Wynn et al., 2004; Hsieh et al., 2006).

In the current study, we wanted to investigate whether a short mindfulness instruction would affect attentional processes as measured within a PPI/PPF paradigm. Two aspects were important. (1) Mindfulness training has been shown to improve attentional processes, but not with such a short mindfulness exposure as utilized in the present study. (2) A PPI/PPF paradigm is ideal to measure attentional processes in such an experiment, as it depends on nonvoluntary measure that requires no intentional motor response. In addition, since it is has the capacity to reveal both inhibition and facilitation, it could reveal both excitatory and inhibitory aspects of processing (Filion et al., 1993). To our knowledge, only one previous study looked at the relationship between PPI and meditative processes (Kumari et al., 2015) finding no increased PPI in expert meditators.

The descriptions of PPI and PPF and the attentional subsystems of alerting and orienting arguably share some similarities. PPI is described as a measure of automatic attention, much in the same way as alerting is defined, whereas PPF is described as a measure of orienting (Graham, 1975) or selective attention (Filion et al., 1993). PPI is, however, most of all considered a mechanism that protects preattentive processing. That is, PPI provides some sort of buffer for arriving information and prevents sensory overload (Braff and Geyer, 1990), which may make it less of a fit with the description of alerting.

Although the overall nature of the current study was exploratory, given a lack of prior research along these lines, the following predictions emerged based on the presented literature. Given that short mindfulness programs (Jha et al., 2007) and early phases of training (Chiesa et al., 2011) both enhanced orienting, we expected that a short mindfulness instruction would enhance PPF. The effect of mindfulness on PPI was more uncertain. For one, only comprehensive MT seems to affect alerting, and Kumari et al. (2015) found no increased PPI in expert meditators. In addition, the link between PPI and alerting is, as mentioned, not that clear.

In the present study, the effect of a short mindfulness instruction on PPI and PPF was investigated. Participants in the mindfulness group listened to a 23-min mindfulness instruction, preceded with a PPI/PPF pretest, and followed by a PPI/PPF posttest. The control group listened to calm classical music for 23 min.

## Materials and Methods

### Participants

Thirty-seven people (12 men, 25 women, age range 20–41, mean age 24.3 years) participated in the study. Five additional participants were excluded from the study because of small startle responses. The participants were randomly assigned to one of two groups (mindfulness or control), the first containing 17 (6 men, 11 women, age range 20–41, mean age 25.1 years) participants and the latter 20 (6 men, 14 women, age range 20–32, mean age 23.6 years). The difference in age between the groups was not significant (*F* < 1.3). All participants reported good health and did not report any hearing problems, previous serious disease, or injury. The participants were instructed to not drink caffeinated beverages and not use nicotine-containing substances for 3 h prior to the start of the study. Written informed consent was obtained from all participants, who were given two lottery tickets (equivalent to 50 NOK) for their participation or course credit for an introductory psychology class.

### Apparatus and Stimuli

The experiment took place in an electrically and acoustically shielded chamber where the temperature was kept at about 20°C. A Bruel and Kjær 2235 Sound Level Precision Meter was used to measure the intensity of auditory stimuli. Programs for experimental control were written in Coulbourn Human Startle System HSW v. 7.500 – 00 and run on a Microsoft Windows XP based Dell PC that controlled presentation of experimental stimuli and data acquisition.

Startle-eliciting noise had an intensity of 95 dB (SPL), instantaneous rise time, and a duration of 50 ms. The prepulses were 1,000 Hz tones with intensity of 60 dB (SPL) and rise time of 10 ms, with a duration of 60, 120, and 2,000 ms. The stimuli were delivered through Sennheiser HD 250 headphones.

Startle eyeblink electromyographic (EMG) responses were recorded from the right orbicularis oculi with two sintered-pellet silver chloride AgCl miniature electrodes (4 mm diameter) filled with Microlyte electrolite gel (Coulbourn Instruments). Inter-electrode distance was 1.0–1.5 cm. The ground electrode was placed centrally on the forehead. The EMG signal was amplified by a factor of 50,000 and filtered (passing 8–1,000 Hz) by a Coulbourn V75-04 bioamplifier. The signal was rectified and integrated with a Coulbourn V76-24 contour-following integrator with a 10-ms time constant, and the output was sent to the PC *via* a LabLinc V interface. A 12 bit A/D board was used. Sampling on each trial began 100 ms prior to onset of the startle stimulus and continued for 200 ms after onset of the startle-eliciting stimulus. The sampling rate was 1,000 Hz.

### Procedure

After arrival at the laboratory the subjects sat down in a desk chair and read and signed the Informed Consent Form. Thereafter, the participants were lead into the experimental chamber and seated in a reclining chair. The subjects were informed of the general purpose of the study and about the stimuli and procedure. They were not informed of any hypothesis for the results. They were told that they could withdraw from the study without giving any reason at any time. The skin below the participants’ right eye was cleaned with a swab containing alcohol and pumice, and the electrodes for measurement of the startle blink electromyography (EMG) were attached. The nature of the stimuli was explained, and participants were told to count the number of tones they heard as a measure of concentration. The headphones were attached, and the experimental procedure was initiated. The door to the experimental chamber was closed during all stimulus presentation.

In the PPI/PPF phase, startle-eliciting noise was presented at lead intervals of 60, 120, and 2,000 ms relative to onset of the prepulse tone. Each lead interval was presented five times. The startle-eliciting noise was also presented 10 times alone (five times at the beginning to reduce the effect of habituation and five times intermingled with the other trials to serve as baseline [S2]), such that a total of 25 trials were presented in the startle phase. The lead intervals were presented in a semirandom order. The order of the lead intervals was 2,000, 120, S2, 60, 120, S2, 60, 120, 2,000, S2, 2,000, S2, 60, 2,000, S2, 120, 60, 120, 60, and 2,000.The ITI was between 17 and 22 s (mean 19 s).

Immediately after the PPI/PPF phase, the manipulation phase was initiated. In the manipulation phase, the mindfulness group listened to a 23 min mindfulness instruction audio tape. The control group listened to calm classical music for 23 min. During the manipulation, the participants sat in a reclining chair, alone in the experimental chamber. The audio was delivered *via* headset. No instruction was given in relation to keeping their eyes open or closed, or other aspects, apart from the instruction to listen to the audio for the entire duration.

After the manipulation phase, the PPI/PPF posttest phase was initiated. This phase was identical to the first PPI/PPF phase.

After the first phase of the experiment the participants received and filled out a schema with for VAS for concentration level. The text read said: “Rate on the line below your level of concentration,” and the endpoint was labeled “Very concentrated” and “Very little concentrated.” The schema also included a space for indicating the number of tones they had heard.

After the second (manipulation) phase, the participants received and filled out the VAS test for concentration again. In addition, they were asked on another VAS to indicate the level of effect they thought the manipulation had: “Rate on the line below how much effect you thought listening to the CD had.” and the endpoints was labeled “Small effect” and “Large effect.”

After the third phase (PPI/PPF posttest) the participants received and filled out a schema with the VAS tests for concentration and manipulation effect, in addition to indicating number of prepulses heard.

### Intervention and Control Condition

Participants in the mindfulness group listened to a 23-min mindfulness instruction, consisting of invitations to pay attention to the moment-to-moment experience of breathing, bodily sensations, thoughts, and emotions with a curious, non-judgmental, and non-striving attitude. The audio file was made by a MBSR-instructor trained by the Center for Mindfulness, Massachusetts. The control group listened to calm classical music. Both conditions were preceded by a PPI/PPF pretest and followed by a PPI/PPF posttest.

### Response Scoring and Data Reduction

Startle blink reflexes were scored as the difference between the maximum amplitude of the EMG response in the window from 0 to 200 ms after noise onset, compared to the mean EMG level for the last 100 ms prior to onset of the startle eliciting noise on that trial. Participants who had a mean score of less than 30 A/D-units above baseline on startle alone in the PPI/PPF pretest were defined as non-responders and deleted from the data set (five participants). Modulation of startle reflexes was calculated as a proportional change from startle reflexes elicited alone (Blumenthal et al., 2004). For this measure, average responses in each stimulus condition were calculated across the entire session. Average responses to the startle-eliciting noise alone (i.e., the control condition) were subtracted from that of each lead interval. This difference was divided by average responses to the startle-eliciting noise alone. A ratio of 0 indicated no modulation of startle reflexes, whereas a ratio above or below 0 meant that the reflex was potentiated or inhibited, respectively. This method is not affected by differences in control startle magnitudes and is termed “proportion of difference from control” (PoD).

Outliers in the startle data (responses 3 SD’s or more from the mean) were replaced by the mean for that participant in that condition. This pertained to less than 1% of all responses.

Tone counting was calculated as percent correct, where 15 was 100% and for every digit away from this number 6.66% was subtracted from 100, with a minimum score of 0. That is, reporting 15 tones was 100% correct, and, e.g., 14 and 16 tones was 93.3% correct.

### Design and Statistics

The design for the experiment was a 2 Group (mindfulness, control) × 3 Lead interval (60, 120, and 2,000 ms) × 2 Phases (pretest and posttest) mixed design where the first factor was treated as a between-subjects factor and the last two factors were treated as within-subjects factors. Analysis was conducted in Statistica 13 analytics software package. Theoretically interesting significant main effects or interactions were followed-up by planned comparisons Least Squares means contrast analysis. The sequential Bonferroni procedure was used to control for multiple comparisons. For ANOVAs involving more than one degree of freedom the uncorrected degrees of freedom, the corrected value of *p* and the epsilon value of the Geiser-Greenhouse were reported.

## Results

### Startle

#### Baseline Data

The analyses of startle to noise alone revealed no significant differences. There was no main effect of Group or Phase, nor interaction of the Group by Phase [*F*(1, 35) = 0.61, *p* = 0.81, ƞ^2^ = 0.02].

#### Complete Model

**Figure 1:**
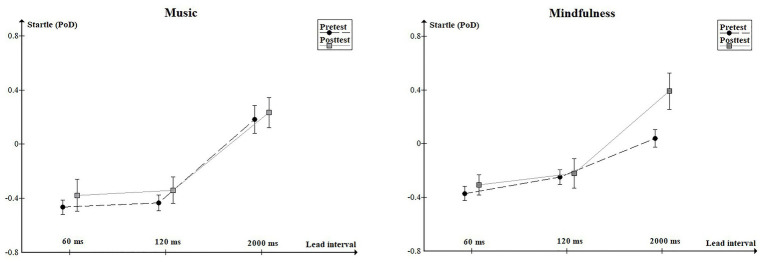
Startle response (as Proportion of Difference from control) at Lead Intervals of 60, 120, and 2,000 ms. Error bars represent +1/−1 SEM.

The Group by Lead interval by Phase repeated measures ANOVA revealed the following results. The main effect of Lead interval was significant [*F*(2, 70) = 69.98, *p* < 0.1, ɛ = 0.70, *η*_p_^2^ = 0.67]. The main effect of Phase was not significant but there was a trend toward greater startle in the posttest [F(1, 35) = 3.21, *p* = 0.08, ɛ = 1.00, *η*_p_^2^ = 0.08]. The interaction of Group by Lead interval by Phase was significant [F(2, 70) = 3.46, *p* < 0.05, ɛ = 0.84, *η*_p_^2^ = 0.09]. No other main effects or interactions were significant. Planned comparison contrast analysis showed greater startle at the 2,000 ms lead interval in phase two (posttest) in the mindfulness group compared to phase one [pretest; *F*(1, 35) = 6.45, *p* = 0.016; Figure 1]. There were no other significant differences between pretest and posttest (*F*s < 1.25 and *p*s > 0.027; see Table 1).

**Table 1:** Follow-up tests of the significant Group by Lead interval by Phase interaction.

Least Squares means contrast analysis		
**Mindfulness group**		
Posttest vs. Pretest	*F*	*p*
60 ms	0.387	0.538
120 ms	0.096	0.758
2,000 ms	6.447	0.016^*^
**Music group**		
Posttest vs. Pretest		
60 ms	0.833	0.368
120 ms	1.253	0.271
2,000 ms	0.148	0.703

* p < 0.05

#### Bayesian Analysis

In order to investigate the lack of difference (at the 60 and 120 ms Lead interval) between pretest and posttest in the mindfulness group we did a Bayesian Repeated Measures ANOVA. The interaction of Group by Lead interval by Phase, which included only the 60 and 120 ms Lead interval, revealed that the null hypothesis was 2.96 times more likely than the alternative hypothesis (BF excl = 2.96). Comparing the models the results showed that the null model was approximately five times more likely than any other model (BF m = 4.89).

### Subjective Data

#### Tone Count

There was a main effect of Phase in the number of tones reported after the pretest or the posttest [*F*(1, 35) = 10.43, *p* < 0.01, *η*_p_^2^ = 0.23], where the participants reported more tones in the posttest. There was no main effect of group, and no interaction of group by phase.

#### Tone Estimation

When tone count was re-calculated to a percent score of correct estimations (where 15 tones was 100%, and, e.g., 14 and 16 tones was 93.3%) there was a main effect of phase [F(1, 35) = 10.43, *p* < 0.01, *η*_p_^2^ = 0.23], where the participants reported more correct number of tones in the posttest. There was no main effect of group, and no interaction of group by phase.

#### Concentration

There were no significant main effects or interactions in reported concentration.

#### Effectiveness

There was no significant main effects or interactions in reported effectiveness.

## Discussion

The main finding in the present study was the increased startle at the 2,000 ms lead interval in the mindfulness group after the mindfulness instruction compared to before the instruction. This increased prepulse facilitation effect was not evident in the control group who had listened to calm classical music. There was no difference at the shorter lead intervals and, hence, no difference in prepulse inhibition following the manipulation. As such, this experiment showed that a short mindfulness manipulation increases prepulse facilitation but not prepulse inhibition.

Jha et al. (2007) found mindfulness-based stress reduction to improve concentrative attention. Concentrative attention is linked to the ability to focus attention on one object such as the breath. In the present study, the participants were asked to count the number of prepulses, thereby focusing attention on one object. Hence, increased focus on the prepulse was expected to increase prepulse facilitation. This is in line with the idea that prepulse facilitation is an index of the attentional ability of *orienting*.

There was no effect of mindfulness on prepulse inhibition, and the Bayesian analysis showed that the null hypothesis was about three times more likely than the alternative. There are several possible explanations of this. For one, it could be that PPI is not linked to an attentive process improved by mindfulness training. Second, it could be that the short instruction utilized in the present study was not sufficient for an effect on PPI to manifest. Third, if PPI is related to the attentional skill *alerting*, one may expect that MT would increase PPI, but only in higher amounts of training, or in expert practitioners. This proposition should be investigated further in future research. A study by Kumari et al. (2015) examined differences in PPI among non-meditators and meditators with several years of experience. They found that meditators showed better performance on attentional tasks despite similar attentional modulation of PPI. This may indicate a stronger attentional capacity, but the authors hypothesized that this concerns more conscious processes of attention than those involving prepulse inhibition.

There were no differences between the two groups on any measures of subjective attention. Both groups performed similarly on tone counting and reported similar levels of concentration and effectiveness. This could point toward a limited effect of the manipulation, and the fact that the mindfulness group did not perform any better on tone counting limits the weight of the results. However, it may also be that the tone-counting task was not sensitive enough to pick up on any difference in attention.

Former research has linked PPI to executive function or conflict monitoring. Bitsios et al. (2006) and Giakoumaki et al. (2006) found that participants who showed more PPI performed better on tasks that involve supervisory attention systems. Dividing participants into “high” and “low” PPI individuals based on startle responses to pulses (with 80 ms prepulse to pulse intervals), they found that the “high” PPI individuals performed better on tests of planning (Giakoumaki et al., 2006), strategy formation, and execution time (Bitsios et al., 2006). Based on these results one could expect modification in PPI if mindfulness increases executive functioning. However, the short mindfulness instruction in the present study was probably not enough to enhance executive functioning.

In their review, A. Chiesa et al. (2013) suggested that mindfulness is related to “top-down” regulations of emotions in short term practitioners, and “bottom-up” emotion regulation in experienced practitioners. This is in line with Corbetta et al. (2002) who proposed segregated systems, where orienting is driven by a bilateral dorsal frontoparietal “top-down” system, and alerting is driven by a right-lateralized ventral frontoparietal “bottom-up” system. Although emotional regulation and attention are different processes altogether, it is interesting to note the correspondence between short-term practitioners, “top-down” processes, and orienting on the one hand, and between experienced practitioners, “bottom-up” processes, and alerting on the other hand.

Taken together these results and the previous research on attentional subsystems seem to have some common features. As we did not measure attention with ANT or other direct measures, we cannot conclude about any links between PPI/PPF and the proposed attentional systems. Future research should include such measures of attention to be able to investigate the possible relationship between PPI/PPF and the specific parts of the attentional system.

There are no available studies known to the authors that have measured the effect of a short mindfulness instruction on prepulse inhibition and facilitation. In the present study, mindfulness instruction for about 20 min was enough to produce a change in PPF. To our knowledge, this is one of the shortest effective manipulations reported. However, Keng et al. (2011) argued in a review of the effects of mindfulness that laboratory studies have shown that it does not take extensive training to see an effect and that some benefits can be seen immediately following mindfulness training. For emotional regulation, Levitt et al. (2004) reported that a 10-min audio rationale for either suppressing or accepting one’s emotions decreased participants’ anxiety during an aversive CO_2_-challenge. Concerning attention, Zeidan et al. (2010) found an effect of four sessions of about 20-min training on sustained attention. One session of about 20 min puts the present study firmly in the same category of short and effective manipulations.

Different methodological challenges are likely to affect research on mindfulness-related improvements of attention. Jensen et al. (2012) call for investigations with the use of attentional measures less confounded by attentional effort or individual test motivation. Others have addressed the issue of varying application of both definition and types of mindfulness used in various studies (Baer, 2003; Bishop et al., 2004). Antonova et al. (2015) underlined the importance of employing experiments with randomized group distribution, pointing to the problem of separating trait and state while using trained meditators as these may have been more prone toward meditation because of a mindfulness trait. These concerns can be avoided in research using psychophysiological measures such as PPI and PPF as part of the paradigm, and in addition using mindfulness naïve participants who are randomized into the different experimental groups.

The present study had some limitations. First, we did not measure the participant prior experience with mindfulness or mediation. It could be that some of the participants had prior experience and this could have affected the results. However, as participants were randomized to the mindfulness/music group, the level of experience was hopefully evenly distributed between the groups. Second, the control condition was not perfect. Ideally, the control group should have been listening to some sort of sham mindfulness instruction. Since we believe this is hard to make, we opted for classical music as control condition. This has been done in other mindfulness studies (e.g., Gu et al., 2018). Third, the experimenter was not completely blind to the hypothesis. There was an expectation of an effect of the manipulation in the mindfulness group. However, we do not believe that any experimenter expectation could have affected the startle data. If there was an effect of experimenter expectation this would have more likely influenced the subjective data, as these are voluntarily controlled by the participants. In fact, the inability to subjectively affect the startle response is, as mentioned, a key strength in using a PPI/PPF paradigm in research.

The startle data showed some deviations from normality, which is not uncommon for startle measurements. This should be noted, as deviations from normality can lead to false positives (see, e.g., Mair and Wilcox, 2020). Earlier, the proposed workaround to this (for startle data) has been to transform the raw data into logarithms, which causes problems of its own (Blumenthal et al., 2004). A better solution for future research would be to use the robust statistics described by Mair and Wilcox (2020). However, we do not know of any reason that normality deviations would lead to a significant result in the 2,000 ms lead interval for the mindfulness group but not the control group. All the same, the deviation of normality should be noted and taken into account when evaluating the conclusions of the present study.

Another potential problem with the experiment was that the mindfulness group did not seem to have prepulse facilitation at the 2,000 ms lead interval in the pretest. Inspecting the figure, the level of response in the pretest seems close to the zero, indicating no difference from the response to startle alone. However, there was no significant difference between the mindfulness group and the control group at this lead interval either. We could only speculate to the reasons for such a small response to the 2,000 ms lead interval in the pretest for the mindfulness group. Nevertheless, the advantage of the within-subjects designs is that we can compare the participants in the posttest to themselves in the prestest. This makes it possible to discover effects that in other circumstances could be lost because of individual differences between groups. The point remains that there was significantly more facilitation in the posttest, after mindfulness instruction, than in the pretest.

To summarize, the present study showed enhanced PPF following a brief mindfulness training. There was no effect on PPI, nor on any of the subjective measures of attention. The results are discussed in relation to three functionally and anatomically distinct subsystems of attention termed: *alerting*, *orienting*, and *conflict monitoring* (Chiesa et al., 2011). The notion that PPI is related to alerting and PPF is linked to orienting should be explored further in future research.

## Data Availability Statement

The raw data supporting the conclusions of this article will be made available by the authors, without undue reservation.

## Ethics Statement

Ethical review and approval was not required for the study on human participants in accordance with the local legislation and institutional requirements. The patients/participants provided their written informed consent to participate in this study.

## Author Contributions

OÅ and MJ developed the theoretical idea, planned the experimental protocol, and performed the data analysis. IS developed and produced the mindfulness procedure, while OÅ incorporated this into the final experimental setup and wrote the manuscript with support from MJ and IS. All authors discussed the results. All authors contributed to the article and approved the submitted version.

### Conflict of Interest

The authors declare that the research was conducted in the absence of any commercial or financial relationships that could be construed as a potential conflict of interest.
